# Targeted Supplementation and Nutritional Strategies for Healthy Aging: A Review of Physiological and Molecular Benefits

**DOI:** 10.1007/s13668-026-00776-y

**Published:** 2026-06-03

**Authors:** Jennifer A. Kurtz, K. Michelle Singleton, Ecaterina Vasenina, Ralf Jäger, Drew Gonzalez, Antonella Schwarz, Jonathan Howard, Jose Antonio

**Affiliations:** 1 Sports Nutrition and Performance Laboratory, Division of Kinesiology, University of Wyoming, Laramie, WY USA; 2 Department of Kinesiology, Coastal Carolina University, Conway, SC USA; 3 Human Performance Laboratory, Department of Health Sciences and Human Performance, The University of Tampa, Tampa, FL USA; 4 Increnovo LLC, Whitefish Bay, WI USA; 5 Occupational, Performance, and Nutrition Laboratory, Department of Kinesiology, Sam Houston State University, Huntsville, TX USA; 6 Department of Sport and Exercise Sciences, Barry University, Miami Shores, FL USA; 7 Department of Kinesiology and Wellness, Georgia Highlands College, Floyd County, GA USA; 8 Department of Health and Human Performance, Nova Southeastern University, Davie, FL USA

**Keywords:** Healthy aging, Dietary supplementation, Mitochondrial function, Inflammation and oxidative stress, Musculoskeletal and cognitive health, Longevity strategies

## Abstract

**Purpose of Review:**

Aging is marked by progressive physiological decline driven by chronic inflammation, mitochondrial dysfunction, and impaired metabolic and musculoskeletal resilience. As the global population ages, dietary supplements have gained attention as potential tools to support healthy longevity. This review summarizes current evidence on nutritional compounds that target aging-related pathways, focusing on interventions that influence mitochondrial health, cognitive performance, immune function, metabolic regulation, and maintenance of muscle mass in older adults. Thirty-two human studies were identified through PubMed, Scopus, and Web of Science.

**Recent Findings:**

Evidence indicates that several targeted nutrients, including protein, probiotics, antioxidants, and emerging mitochondrial-support compounds, may contribute to healthy aging. Protein and collagen supplementation, particularly when paired with resistance training, consistently improve muscle mass and physical function. Antioxidants, glycine, N-acetylcysteine, and NAD⁺ precursors show potential benefits for mitochondrial efficiency, cellular stress responses, and cognitive performance; however, findings vary across populations and dosing strategies. Heterogeneity in study designs, supplement formulations, and biomarker endpoints underscores the need for individualized and context-specific application.

**Summary:**

A personalized, evidence-informed supplementation strategy integrated with exercise and balanced nutrition may help optimize physiological function in aging adults. Future research should refine dosing protocols, evaluate synergistic nutrient combinations, and clarify the roles of antioxidants and mitochondrial-targeted compounds across diverse older populations.

## Introduction

Aging is a complex, multifaceted process marked by a gradual decline in physiological.

function across all systems of the body, including metabolic, cardiovascular, musculoskeletal, immune, and cognitive domains [1, 2]. Functional declines are driven by biological mechanisms such as genomic instability, mitochondrial dysfunction, cellular ageing, and chronic low-grade inflammation [1, 3]. Importantly, these biological changes manifest most meaningfully through declines in functional capacity, including reduced muscle strength, impaired metabolic regulation, diminished cognitive performance, and increased disease risk. As life expectancy increases worldwide, the emphasis is shifting from simply prolonging life to enhancing the quality of life during aging.

Dietary supplementation has emerged as a promising strategy to support healthy aging by targeting specific biological pathways that contribute to age-related decline [4]. Nutrients and bioactive compounds such as antioxidants, NAD+ precursors, mitochondrial cofactors, anti-inflammatories, and immune-modulating agents have been investigated for their potential to preserve cellular function, enhance metabolic health, and delay the onset of chronic diseases [5, 6]. However, the breadth of outcomes studied in the supplementation literature varies widely, ranging from clinically meaningful functional endpoints to surrogate or cosmetic outcomes, which can obscure translational relevance to healthspan. This narrative review adopts a focused framework centered on healthspan-relevant functional outcomes rather than aesthetic or purely surrogate endpoints. This article aims to synthesize evidence from human studies evaluating dietary supplements that directly or indirectly modulate the recognized hallmarks of aging, such as mitochondrial dysfunction, oxidative stress, inflammation, and proteostasis. Primary emphasis is placed on outcomes that reflect functional aging trajectories, including musculoskeletal function, metabolic health, cognitive performance, and immune resilience. Rather than providing an exhaustive account of all supplements ever studied, this review focuses on representative compounds with the strongest or most emerging human evidence.

The purpose of this review is to examine current evidence surrounding anti-aging supplements and their impact on functional and physiological outcomes directly linked to health span. The goal is to provide a focused and integrative synthesis of dietary strategies that promote healthy aging and longevity, emphasizing compounds that support mitochondrial function, cognitive performance, immune defense, musculoskeletal strength, and metabolic balance. By prioritizing clinically meaningful endpoints and clearly distinguishing them from cosmetic or surrogate outcomes, this review aims to improve conceptual clarity and enhance translational applicability for both researchers and practitioners.

## Methods

This narrative review was conducted to synthesize current evidence on dietary supplements that support healthy aging. A comprehensive literature search was performed using PubMed, Scopus, and Web of Science to identify peer-reviewed articles published between 2010 and 2025. Search terms included combinations of “anti-aging supplements,” “healthy aging,” “mitochondrial health,” “cognitive function,” “immune support,” “muscle mass,” “collagen,” “protein supplementation,” “NAD+ precursors,” “GlyNAC,” and “oxidative stress.” For the purposes of this review, “hallmarks of aging” is defined according to established frameworks [1] and included mitochondrial dysfunction, loss of proteostasis, deregulated nutrient sensing, cellular senescence, and chronic low-grade inflammation. These hallmarks guided the categorization of physiological outcomes (e.g., muscle preservation, metabolic regulation, and immune resilience) examined across included studies. Inclusion criteria were studies involving adult human participants and evaluating the effects of dietary supplementation on aging-related physiological outcomes; animal studies were included selectively to provide mechanistic insights. Studies focused primarily on clinical pathologies unrelated to aging (e.g., COPD, cancer cachexia, or metabolic disorders) were excluded unless outcomes directly informed aging-related mechanisms (e.g., mitochondrial function, muscle, or cognition). While studies with very small sample sizes (*n* < 10) were generally excluded, this criterion was applied contextually rather than as an absolute threshold. Select pilot or proof-of-concept studies (e.g., GlyNAC interventions) were included when they provided unique mechanistic insight or addressed underrepresented populations and are interpreted accordingly as preliminary evidence. Studies were also excluded if they had low sample sizes (generally *n* < 10, unless contextually justified for mechanistic or pilot data), lacked control groups, or did not report relevant biomarkers or performance outcomes aligned with the defined hallmarks of aging.

Titles and abstracts were screened for relevance, followed by full-text evaluation based on inclusion criteria. Reviews, clinical trials, and observational studies were included if they contributed mechanistic or applied insight into aging-related pathways. Each team member independently researched specific physiological domains (e.g., mitochondrial function, cognitive health, immune resilience, musculoskeletal support, and metabolic balance), and findings were compiled into a comprehensive draft. This draft was then reviewed and refined by the lead authors to ensure accuracy, consistency, and alignment with current scientific understanding. Studies were evaluated using a structured evidence-informed framework. Evidence was qualitatively categorized as “strong” (well-powered randomized controlled trials or consistent findings across multiple human studies), “moderate” (smaller randomized trials or well-designed observational studies), or “emerging” (pilot trials, mechanistic studies, or limited human data). This framework was used to guide interpretation and ensure that conclusions were aligned with the strength and consistency of the available evidence.

Several recent studies were excluded from this review due to small sample sizes, short interventions, heterogeneous dosages, or outcomes not directly related to aging biology. Many antioxidant RCTs also differ in baseline nutritional status, delivery method, or population studied, limiting consistency in synthesis [7, 8]. For example, NAD⁺ precursors like NMN, compounds such as NR and MitoQ were excluded due to limited or inconsistent evidence in aging-specific cohorts. Emerging findings such as quercetin’s effects on telomere length [9], and ongoing challenges with its bioavailability [10] illustrate both the promise and complexity of antioxidant supplementation. Where smaller or exploratory studies are discussed, conclusions have been explicitly tempered to reflect their preliminary nature and to avoid overinterpretation. These exclusions reflect a deliberate scope refinement to ensure the review remains methodologically rigorous, evidence-informed, and clinically relevant.

## Results

A total of 21 studies met inclusion criteria and were summarized to evaluate the impact of dietary supplementation on aging-related outcomes in older adults. Interventions ranged from macronutrient-based supplements (e.g., whey or collagen protein) to targeted bioactive compounds including probiotics, NAD+ precursors, and antioxidant combinations such as GlyNAC. While results varied by supplement type and study design, several interventions demonstrated beneficial effects on muscle mass, strength, immune modulation, cognitive performance, gut health, and markers of metabolic health. The most robust effects were observed in multimodal interventions combining resistance training with protein supplementation and in studies utilizing compounds that directly support mitochondrial and cellular function (Table 1).

**Table 1:** Summary of studies exploring the effects of dietary supplementations in older adults

Study	Participants	Supplement	Dosage	Timing	Main findings	Evidence Strength
Mertz et al. [11]	208 healthy older adults (*n* = 109 males, *n* = 99 females)	Randomly assigned to 1 of 5 interventions: Carbohydrate (CARB), collagen protein (COLL), whey protein (WHEY), light-intensity exercise with whey protein (LITW), and heavy resistance training with whey supplementation (HRTW).	CARB, COLL, WHEY, LITW, HRTW: 2 daily doses of 20 g supplement + 10 g sucrose (type varied by group).	Supplements taken twice/day; LITW and HRTW groups took one dose post-exercise.	HRTW was the only group effective in preserving both muscle mass and increasing strength.	Strong
Clark et al. [12]	147 participants competed on a varsity team or club sport (*n* = 72 males, *n* = 75 females)	Participants were randomly assigned to two groups. One group received a liquid formulation containing collagen hydrolysate and the other group received a liquid containing xanthan.	Treatment: 10 g collagen hydrolysate (25 mL); Placebo: xanthan solution (25 mL).	Participants consumed the liquid formation for 24-weeks.	Collagen hydrolysate can aid in supporting joint health and suggests the supplement may reduce joint deterioration.	Weak-Moderate
Walden et al. [13]	30 healthy, older women (58.5 ± 5.2 years)	Participants were supplemented with rice and pea protein concentrate blend. *Weizmannia coagulans* (BC30) in the spore form was added to each protein dose. For the placebo arm of the study, maltodextrin was added. .	Participants received 27 g rice/pea protein (20 g protein) with or without 1 × 10⁹ CFU BC30; placebo matched in mass.	Daily 2-weeks.	Addition of BC30 to a daily 20 g dose of plant protein concentrates may improve the appearance of amino acids in the blood.	Strong
Probiotic Supplementation[1–3, 14–16]	Older adults (*n* ≈ 50–100 per study)	Multi-strain probiotics (e.g., *B. longum*, *L. helveticus*, *L. plantarum*)	~ 10⁹ CFU/day	4–12 weeks	Probiotic supplementation shows preliminary support immune function, gut microbiota composition, and aspects of cognitive and mood-related outcomes in older adults; however, findings are heterogeneous and appear strain-, duration-, and population-specific.	Weak-Moderate
Kumar et al[17, 18]	Older adults (OA, *n* = 32) and young adults (YA, *n* = 20)	GlyNAC (glycine + N-acetylcysteine) supplementation ± alanine placebo	Glycine 1.33 mmol/kg/day, NAC 0.81–100 mg/kg/day	16–24 weeks; OA follow-ups at 4, 8, 12 weeks; YA 2 weeks	GlyNAC reduced oxidative stress, improved glutathione and mitochondrial function, decreased inflammation, improved endothelial function, physical performance, strength, gait, cognition, and body composition in older adults.	Moderate
Yoshino et al. [19]	25 postmenopausal women with prediabetes and who were overweight or obese	12 participants were randomized to the placebo group and 13 were randomized to the nicotinamide mononucleotide (NMN) group.	NMN participants received 250 mg/day.	10 weeks.	NMN improved muscle insulin signaling, insulin sensitivity, and remodeling in postmenopausal women with prediabetes.	Weak-Moderate
Timmers et al. [20]	11 obese, healthy males	Participants participated in two experimental trials, a placebo and a resveratrol condition.	Resveratrol dose consisted of 150 mg/day.	Daily for 30 days.	Resveratrol for 30 days improved metabolic profiles in healthy obese males, reducing liver fat, glucose, triglycerides, ALT, and inflammation.	Moderate
Ahmadi et al. [21]	42 patients with metabolic syndrome (MetS) (*n* = 12 males, *n* = 30 females)	Participants were randomly divided into the alpha lipoic acid (ALA) or placebo groups. Each group consisted of 23 participants.	ALA group received 600 mg ALA per capsule; placebo contained starch.	12-weeks.	ALA improved triglycerides and total cholesterol, with no change in HDL-C or LDL-C, in patients with MetS.	Moderate
Liu et al[22], Nakagawa et al[23], Satoh et al[24]	Adults and older adults (*n* = 209; ages 20–69)	Astaxanthin supplementation (± tocotrienol & zinc in Liu) or astaxanthin-rich Haematococcus pluvialis oil	4–20 mg/day	4–16 weeks	Astaxanthin supplementation may support muscle strength, endurance, mobility, erythrocyte antioxidant status, metabolic markers, and aspects of cognitive function. Cognitive and metabolic effects are preliminary and should be interpreted cautiously.	Moderate-Emerging
Dickerson et al[25]; Chiu et al[26], Stiefvatter et al[27]	Sedentary healthy women (*n* = 71) and adults aged 40–75 years (*n* = 44)	Microalgae-derived supplements (fucoxanthin from *Phaeodactylum tricornutum*, Chlorella growth factor, microalgae biomass/extract)	~ 4.4 mg/day fucoxanthin; 27 mL/day CGF; 1.8–2.3 g/day microalgae biomass or supernatant extract	12–24 weeks	Microalgae-derived supplementation may improve antioxidant status, lipid profiles, inflammation, and select functional outcomes (e.g., aerobic capacity, activity tolerance); effects on body composition are inconsistent and vary by compound and population.	Emerging
Koite et al[28]; Ismail et al[29]	Adults with metabolic syndrome (*n* = 40) and COPD patients (*n* = 30; plus 20 controls)	Spirulina/*Arthrospira*-based supplementation	~ 20 mg/day (Spirulysat^®^) to 500–1000 mg/day spirulina	8–12 weeks	Spirulina may improve oxidative stress and some lipid markers, but effects are population-specific and not consistent across all cardiometabolic outcomes.	Emerging-Moderate
Sugawara et al[30]; Pungcharoenkul & Thongnopnua[31]	Sedentary postmenopausal women (*n* = 45) and healthy adults (*n* = 24)	Curcumin supplementation ± exercise (vs. placebo or vitamin E)	25 mg/day (Sugawara); 500 mg–6 g/day (Pungcharoenkul)	1–8 weeks	Curcumin supplementation may improve cardiovascular function (reducing LV afterload), and lipid profiles (cholesterol, triglycerides); combined with exercise, effects may be greater.	Moderate
Liu et al. [22]	66 adults between the ages of 65 and 90 years (*n* = 16 males, *n* = 50 females)	Participants were randomized to either urolithin A (Uro-A) or placebo intervention groups.	All participants received softgels containing either 250 mg of (Uro-A) along with excipients or only excipients.	4 softgels/day, 16-weeks	Uro-A is beneficial for muscle endurance and plasma biomarkers.	Emerging

## Specific Nutrients

### Protein and Muscle Maintenance

The effects of dietary protein and protein supplementation on older adults has been examined regarding skeletal muscle mass and strength, physical performance, and appetite regulation. In a systematic review and meta-analysis by Kim et al., they concluded that higher protein diets assisted older adults in retaining lean body mass as well as promoting the loss of more fat mass during weight loss [32]. On the other hand, protein supplementation alone does not significantly increase lean body mass or muscle strength in nonfrail older adults unless combined with resistance training [11, 33]. In the trial by Mertz, et al., 208 older adults (> 65 years) were randomly assigned to one of five groups: carbohydrate supplementation, collagen protein (COLL), whey protein (WHEY), light-intensity resistance training with whey, or heavy resistance training with whey. Supplements contained 20 g protein + 10 g carbohydrate (except the carbohydrate group = 30 g carbohydrate) and were taken twice daily. The primary clinical outcome was quadriceps cross-sectional area, with secondary measures including strength, power, function, and body composition. Interestingly, only heavy resistance training with whey preserved skeletal muscle and increased strength [11]. Moreover, protein supplementation can improve gait speed in sarcopenic older adults [34]. Ensuring adequate protein intake is crucial for preventing sarcopenia and promoting musculoskeletal health in the aging population. Older adults are particularly vulnerable to insufficient protein intake. Certainly, the recommended allowance (0.8 g/kg/day) is inadequate due to age-related anabolic resistance [35]. Maintaining skeletal muscle function during aging requires sufficient dietary protein to offset anabolic resistance and support muscle protein synthesis. Evidence from controlled trials and meta-analyses indicates that intakes modestly above the current recommended dietary allowance (RDA; 0.8 g/kg/day), typically in the range of 1.0–1.3 g/kg/day, may better support muscle maintenance in older adults, particularly when combined with resistance training [35–37]. While some studies have explored higher intakes (~ 1.6 g/kg/day), evidence remains inconclusive regarding long-term benefits or safety in all populations. Protein quality also contributes to outcomes, with leucine-rich sources (e.g., dairy, soy, or mixed animal-plant blends) demonstrating greater stimulation of muscle protein synthesis compared to lower-leucine sources. However, balanced and varied protein sources, combined with total protein adequacy and exercise, remain the most consistent determinants of muscle preservation in older individuals.

### Antioxidants and Cellular Protection

Antioxidant supplementation plays a crucial role in cellular protection and aging by mitigating oxidative stress and inflammation, supporting mitochondrial function, and reducing inflammation. Long-term antioxidant supplementation has also been associated with healthier aging in men [38]. Carotenoids are potent antioxidants that help protect against aging by neutralizing free radicals, reducing oxidative stress, and supporting cellular health [39–42]. Compounds such as astaxanthin, lutein, zeaxanthin, and beta-carotene have shown benefits for skin, eye, and cognitive health, and may reduce inflammation and age-related decline [39–42]. For example, astaxanthin helps counter skin aging by boosting collagen production, improving moisture retention, and reducing wrinkles through its strong antioxidant effects [43]. Lutein and zeaxanthin support vision and have been linked to better cognitive function, including improved memory and neural efficiency in older adults [44]. Beta-carotene, a vitamin A precursor, supports immune health and may support cellular redox balance in neurodegenerative diseases like Alzheimer’s and Parkinson’s [45, 46]. Carotenoids also reduce inflammation by downregulating pro-inflammatory cytokines such as TNF-α and IL-6, which are associated with cellular aging and chronic disease [47, 48]. However, research on carotenoid supplementation is mixed. Some studies show limited or no benefit, possibly due to differences in dosage, absorption, or individual metabolism [49, 50]. Although generally safe, high doses, especially of beta-carotene, have been linked to adverse effects, including an increased lung cancer risk in smokers and possible pro-oxidant effects, raising concerns about long-term use in some groups [51]. These safety considerations highlight the importance of evaluating antioxidant supplementation on a case-by-case basis, particularly when transitioning to other fat-soluble antioxidants like vitamin E.

Vitamin E, a fat-soluble antioxidant, protects cell membranes by inhibiting lipid peroxidation, which is crucial for cellular integrity and cardiovascular health [52]. It helps reduce oxidative modification of low-density lipoprotein (LDL), a key factor in atherosclerosis, potentially lowering cardiovascular disease (CVD) risk [53]. Some studies suggest vitamin E may improve endothelial function, reduce inflammation, and support immune function, though evidence for primary CVD prevention remains inconsistent [54–56]. Vitamin E, which includes tocotrienols and tocopherols, helps prevent lipid peroxidation and supports heart and brain health. Its intake has also been linked to slower cellular aging [57]. Its antioxidant properties may delay neurodegeneration in disorders like Alzheimer’s and Parkinson’s disease, though its therapeutic use remains debated [58, 59]. Observational data links higher vitamin E intake to slower cognitive decline in older adults [60]. It also interacts with other antioxidants, such as glutathione, selenium, vitamin C, and carotenoids, which may enhance cognitive performance, although more research is needed to confirm these effects [60, 61]. Additionally, vitamin E may support mitochondrial function and reduce inflammation, both linked to aging and longevity. However, clinical trials on cognitive benefits show mixed results, and concerns exist about high-dose supplementation, which may increase all-cause mortality and hemorrhagic stroke risk [54, 62]. Variability in outcomes likely reflects differences in isoforms, baseline nutrition, and antioxidant interactions. A tailored approach to supplementation is essential, as the overall benefits and risks of vitamin E in aging and disease prevention remain inconclusive.

Selenium is an essential micronutrient that supports immune function and reduces age-related inflammation through its role in selenoproteins such as glutathione peroxidases and thioredoxin reductases. These proteins regulate redox signaling, strengthen antioxidant defenses, and control inflammation[63–65]. Selenium supports immune function by maintaining cellular integrity and regulating inflammatory responses. It has been linked to a reduced risk of cognitive decline and certain cancers[66, 67]. As a cofactor for glutathione peroxidase, selenium plays a key role in immune cell activation, differentiation, and proliferation, while helping to prevent excessive inflammatory signaling. It modulates pathways such as MAP kinase, reducing the expression of pro-inflammatory mediators including TNF-alpha and COX-2 [66, 67]. Further, selenium may contribute to a lower risk of cardiovascular disease and neurodegeneration by limiting chronic low-grade inflammation. It also supports genomic stability by reducing DNA damage and facilitating the removal of misfolded proteins and reactive byproducts [63, 68]. Additionally, selenium is associated with cellular longevity, in part through the preservation of telomere length, which is closely linked to aging and disease risk [63]. Supplementation has been linked to lower levels of inflammatory biomarkers, potentially reducing cardiovascular mortality and improving heart function in older adults [69]. However, research on selenium supplementation shows mixed results. Some studies report no significant effects on inflammation or immune function, likely due to differences in baseline selenium levels, dosage, and duration [7]. High intake may cause toxicity, including selenosis and increased risk of type 2 diabetes in some populations [70, 71]. These findings highlight the need for further research to better understand selenium’s benefits and risks, especially in aging populations.

Quercetin, a flavonoid found in various fruits, vegetables, and teas, has gained attention for its potential anti-aging effects due to its senolytic properties, which help clear senescent cells, dysfunctional cells that accumulate with age and contribute to chronic inflammation and tissue dysfunction [72]. By inducing apoptosis in these cells, quercetin reduces inflammatory cytokines associated with cellular senescence, mitigating the pro-inflammatory environment linked to aging and disease [73]. Beyond its senolytic activity, quercetin exerts cardiovascular benefits It enhances endothelial function by increasing nitric oxide bioavailability and reducing LDL oxidation, thereby supporting vascular health [74]. In aging populations, where endothelial dysfunction raises cardiovascular risk, quercetin’s antioxidant and anti-inflammatory actions may help lower the risk of hypertension and atherosclerosis [75]. It also modulates longevity-related pathways, including AMP-activated protein kinase activation and mitochondrial biogenesis [76, 77]. However, inconsistencies in the literature raise questions about its efficacy. While preclinical and some human studies are promising, variability in bioavailability, dosage, and design has led to mixed clinical results [8]. Quercetin’s low oral bioavailability and rapid metabolism limit systemic effects, prompting interest in optimized delivery. Safety concerns also exist with high-dose supplementation (typically > 1,000 mg/day). Potential adverse effects include gastrointestinal discomfort, nephrotoxicity, and interactions with medications such as anticoagulants and chemotherapy drugs [78, 79]. More large-scale, long-term human trials are needed to clarify quercetin’s safety profile and determine effective dosing strategies for aging populations. Antioxidant supplementation, including carotenoids, vitamin E, selenium, and quercetin, offers promising potential in combating aging-related damage by mitigating oxidative stress, reducing inflammation, and supporting cellular health.

### Microbiota-Based Interventions to Support Immune, Cognitive, and Metabolic Health

The human gut microbiota undergoes dynamic changes throughout the lifespan, influenced by dietary, environmental, and lifestyle factors. In older adults, the gut microbiota typically exhibits reduced alpha diversity and altered taxonomic structure. A notable decline in beneficial butyrate-producing bacteria, such as *Faecalibacterium prausnitzii* and *Bifidobacteria*, is commonly observed, along with an increase in pro-inflammatory taxa, including members of the *Enterobacteriaceae* family [80]. Given their anti-inflammatory and immunomodulatory properties, the age-related decline in *Bifidobacteria* is associated with immunosenescence, elevated systemic inflammation, and increased susceptibility to disease. Interestingly, *Bifidobacterium longum* was detected in 100% of centenarians but in only 30% of younger adults, highlighting the potential importance of this specific species in promoting healthy aging [81]. Probiotics help maintain intestinal barrier integrity, which tends to decline with age, contributing to systemic inflammation. By enhancing tight junction proteins and reducing permeability, probiotics reduce microbial translocation and endotoxin-related inflammation. Daily supplementation with a multi-strain probiotic including *Bifidobacterium longum* Bar33 significantly enhanced immune function in elderly individuals (> 75 years), highlighting the immunomodulatory potential of specific probiotic strains in aging populations. Preliminary evidence suggests targeted microbiota interventions may help counteract immunosenescence and systemic inflammation associated with advanced age [14].

In addition to gut-immune interactions, these mechanisms are implicated in cognitive aging. Certain probiotic strains have been shown to increase brain-derived neurotrophic factor (BDNF) levels and enhance the synthesis of neurotransmitters, including serotonin, which can positively influence mood, cognition, and sleep [15]. BDNF, a neurotrophin involved in neuronal plasticity [82], plays a critical role in learning and memory processes [10]. A 12-week supplementation with a multi-strain probiotic containing *Bifidobacterium bifidum* BGN4 and *Bifidobacterium longum* BORI in elderly adults (> 65 years) resulted in increased brain-derived neurotrophic factor (BDNF) levels and improved mental flexibility. Additionally, probiotic supplementation significantly reduced the abundance of pro-inflammatory gut bacteria, including *Eubacterium* and *Clostridiales*. Notably, these changes in gut microbiota were negatively correlated with serum BDNF levels, suggesting a potential link between gut health and brain function [16].

Probiotics improve bowel regularity and reduce gastrointestinal symptoms common in older adults, such as constipation, bloating, and gas. Probiotics also assist in the digestion, absorption and synthesis of crucial nutrients such as protein, vitamin B12, vitamin K, calcium, and magnesium, which are critical for muscle, bone and cardiovascular health in older adults [13]. Overall, probiotic supplementation may support healthy aging by improving gut microbiota composition, enhancing immune and cognitive function, reducing inflammation, and promoting gastrointestinal and nutritional health in older adults.

## Cellular and Mitochondrial Health

### Mitochondrial Decline and Oxidative Stress

Mitochondria, the energy-producing organelles of the cell, are central to maintaining metabolic homeostasis, cellular signaling, and apoptosis regulation. With age, mitochondrial function declines due to accumulated oxidative damage, impaired biogenesis, and reduced availability of key cofactors such as nicotinamide adenine dinucleotide (NAD+) [83, 84]. This decline is further driven by mutations in mitochondrial DNA (mtDNA), chronic oxidative stress, and diminished mitophagy, which together compromise cellular energy production and resilience. Reactive oxygen species (ROS), byproducts of oxidative phosphorylation, can damage lipids, proteins, and DNA when unregulated, perpetuating a self-reinforcing cycle of mitochondrial impairment and further ROS generation [83]. Given the central role of mitochondrial dysfunction and oxidative stress in aging, these mechanisms provide a unifying framework for the functional domains discussed below. In addition to mitochondrial dysfunction, these mechanisms contribute to the onset and progression of numerous age-related conditions, including neurodegeneration, cardiovascular disease, and sarcopenia [1, 85]. As the understanding of mitochondrial dysfunction expands, targeted nutritional and supplemental strategies that enhance antioxidant defense systems, support mitochondrial biogenesis, and preserve mitochondrial integrity have emerged as promising interventions to promote healthy aging.

### GlyNAC Supplementation and Glutathione Restoration

The combination of glycine and N-acetylcysteine (GlyNAC) supports mitochondrial health by replenishing intracellular glutathione (GSH), one of the body’s most powerful antioxidants [86]. Glutathione neutralizes ROS and maintains redox balance, which is critical for mitochondrial energy production. Studies in older adults show that GlyNAC supplementation restores GSH levels, improves mitochondrial function, and enhances physical performance [17, 18]. Long-term supplementation (16–24 weeks) has been shown to improve insulin sensitivity, muscle strength, and cognitive function while reducing oxidative stress and inflammation [17, 18]. Beyond replenishing antioxidants, GlyNAC may stimulate mitophagy and mitochondrial biogenesis, thereby enhancing mitochondrial turnover and renewal [87]. These protective and regenerative effects position GlyNAC as a foundational anti-aging intervention.

### NAD+ Precursors: Nicotinamide Mononucleotide (NMN) and Nicotinamide Riboside (NR)

NAD + is an essential coenzyme for redox reactions, sirtuin activation, and DNA repair, all of which decline with age [88]. Supplementation with NAD+ precursors such as nicotinamide mononucleotide (NMN) and nicotinamide riboside (NR) restores NAD+ levels and promotes mitochondrial resilience. NMN supplementation has been shown to improve muscle insulin sensitivity and mitochondrial respiration in aging adults [19]. Similarly, NR, a vitamin B3 derivative, has demonstrated efficacy in elevating NAD+ levels and improving cardiovascular and metabolic markers. In clinical studies, NR increased NAD+ concentrations by up to 60% and improved skeletal muscle mitochondrial function in older adults [89–91]. By activating sirtuins (particularly SIRT1 and SIRT3), NR and NMN enhance mitochondrial biogenesis, improve oxidative metabolism, and promote DNA repair, mechanisms that collectively slow cellular aging [92–94]. NR supplementation has also been associated with improved endothelial function, reduced arterial stiffness, and enhanced energy metabolism [90]. These findings underscore NAD+ restoration as a cornerstone strategy for maintaining mitochondrial and cellular vitality during aging.

### Mitochondrial-Targeted Antioxidants

Traditional antioxidants may have limited access to the mitochondria, where reactive species are predominantly generated. In contrast, mitochondrial-targeted compounds such as coenzyme Q10 (CoQ10), MitoQ, and alpha-lipoic acid (ALA) are designed to localize within mitochondrial membranes, allowing them to directly support mitochondrial integrity and function.

CoQ10, an essential component of the electron transport chain, facilitates ATP production while protecting mitochondrial membranes from damage [95]. Supplementation has been associated with improved exercise performance, enhanced cardiac efficiency, and reduced fatigue in aging populations [96]. MitoQ, a mitochondria-targeted derivative of CoQ10, accumulates within mitochondria and has been shown to protect against lipid peroxidation and mitochondrial DNA damage, with reported benefits for endothelial function and cognitive health [97, 98]. ALA exhibits complementary effects, functioning as both a mitochondrial cofactor and redox modulator. In addition to regenerating endogenous antioxidants, ALA supports mitochondrial biogenesis through preservation of PGC-1α signaling and improves insulin sensitivity and glucose utilization [99, 100]. Collectively, these compounds converge on a central outcome: preservation of mitochondrial function and energy metabolism, key determinants of healthy aging.

### Dietary Nucleotides and Cellular Repair

Nucleotides are essential for DNA and RNA synthesis, supporting cellular replication, repair, and immune function. Endogenous production may decline with age and physiological stress, potentially limiting tissue maintenance and recovery [101]. Supplementation has been shown to enhance immune function, support mitochondrial DNA replication, and improve cellular turnover [102–104]. These effects are particularly relevant in aging populations, where nucleotide availability may limit tissue repair and immune resilience. Clinical evidence further suggests benefits for T-cell function and reduced apoptosis, indicating a role in maintaining immune resilience and cellular integrity in aging populations [102, 104].

### Dosage and Safety Considerations

Effective dosing varies across compounds, with reported ranges including 100–300 mg/day for NR, 250–500 mg/day for NMN, 600–1200 mg/day for ALA, and 100–200 mg/day for CoQ10 [90, 96]. GlyNAC protocols commonly utilize ~ 100 mg/kg/day of both glycine and N-acetylcysteine [17, 18]. Overall, these interventions demonstrate favorable safety profiles with minimal adverse effects; however, long-term studies are needed to establish optimal dosing strategies and evaluate potential synergistic effects when combined.

### Conclusion

Mitochondrial and cellular dysfunction are central features of aging. Interventions targeting antioxidant defense, NAD⁺ metabolism, and cellular repair, including GlyNAC, NMN, NR, and mitochondrial-targeted compounds, offer a multifaceted approach to preserving metabolic function and health span. Future work should focus on defining synergistic interactions and developing evidence-based protocols tailored to aging populations.

## Cardiovascular and Metabolic Health

Cardiovascular and metabolic health represent two of the most critical determinants of healthy aging. With advancing age, individuals experience progressive declines in vascular elasticity, endothelial function, mitochondrial efficiency, and glucose tolerance. These changes collectively increase the risk of hypertension, atherosclerosis, type 2 diabetes, and metabolic syndrome. Nutritional strategies and targeted supplementation may help counter these declines. Among the most studied compounds are omega-3 fatty acids (EPA/DHA), resveratrol, magnesium, and alpha-lipoic acid (ALA), which act through complementary mechanisms to support vascular integrity, reduce systemic inflammation, and optimize metabolic function.

### Omega-3 Fatty Acids (EPA/DHA)

Omega-3 fatty acids, particularly eicosapentaenoic acid (EPA) and docosahexaenoic acid (DHA), commonly found in oily fish, demonstrate significant anti-inflammatory properties through modulation of key signaling pathways [105–107]. A meta-analysis by Kavyani et al. [107] systematically evaluated omega-3 supplementation in adults with various inflammatory conditions. The results demonstrated that EPA and DHA independently reduced inflammatory markers, combined EPA and DHA supplementation showed superior efficacy, and optimal effects were observed at doses ≥ 2 g/day. In addition to reducing circulating cytokines, omega-3s are incorporated into phospholipid membranes, where they alter lipid raft composition and subsequently influence receptor activity and downstream immune responses. This structural incorporation helps shift the balance toward production of less pro-inflammatory eicosanoids, which is a key mechanism underlying their systemic effects.

The cardioprotective effects of omega-3 fatty acids remain controversial. A 2021 systematic review and meta-analysis by Khan et al. [108] demonstrated significant reductions in cardiovascular mortality and improved cardiovascular outcomes with omega-3 supplementation. Notably, their analysis revealed that EPA monotherapy showed superior cardiovascular risk reduction compared to combined EPA and DHA formulations. Similarly, Yan et al.’s [109] comprehensive meta-analysis of 15 randomized trials found moderate quality evidence for omega-3 fatty acids in reducing the risk of major cardiovascular events. Clinical evidence suggests cautious application of omega-3 fatty supplementation for individuals with previous myocardial infarction, which may increase the risk of stroke [109]. These mixed findings highlight the complexity of fatty acid metabolism, as benefits appear to be influenced not only by dose and formulation but also by background dietary intake, genetic polymorphisms affecting fatty acid desaturases, and co-existing medical conditions.

Beyond these cardiovascular considerations, omega-3 fatty acids serve as crucial structural components of brain cell membranes and play fundamental roles in neurophysiological processes [110–112]. Regular consumption of omega-3 supplementation is beneficial for cognitive and emotional development, reading skills, and concentration abilities [110, 113, 114]. Clinical evidence indicates that therapeutic omega-3 doses between 500 and 2000 mg per day may alleviate symptoms of depression and anxiety, further supporting cognitive health [115]. EPA, in particular, has been linked to modulation of serotonin and dopamine neurotransmission, while DHA is essential for maintaining neuronal membrane fluidity and synaptic plasticity, two features critical for learning and memory. This neurobiological basis helps explain why relatively modest doses can yield measurable benefits in mood and cognition.

Omega-3 fatty acids demonstrate a variety of benefits from reducing systemic inflammation to supporting brain health, though cardiovascular effects require population-specific considerations. While ≥ 2 g/day shows optimal anti-inflammatory effects, lower doses ranging from 500 to 2000 mg/day may suffice for neurocognitive benefits [115]. From a practical perspective, dosing strategies should be individualized, as the optimal intake likely depends on baseline health status, dietary habits, and specific physiological goals.

### Resveratrol

Resveratrol is a naturally occurring polyphenol (a type of antioxidant) found in foods such as grapes, berries, wine, peanuts, and soy [116]. This bioactive molecule has attracted significant research attention due to its demonstrated potential to modulate aging processes and mitigate age-related pathologies. A key mechanism involves activation of sirtuin 1 (SIRT1), a protein that aids in regulating critical cellular processes, commonly referred to as the “longevity pathway”. While animal studies consistently demonstrate resveratrol’s ability to extend lifespan, human trials show more variable results, likely due to differences in bioavailability and optimal dosing [117]. Resveratrol supplementation may also act as a caloric restriction mimetic in humans [20], replicating the metabolic benefits of fasting or reduced calorie intake [118] further leading to improved insulin sensitivity, lowering body fat, and increasing lipid mobilization in adipose tissue [119]. These multi-system effects position resveratrol as a viable therapeutic strategy for obesity management in aging.

Beyond metabolic benefits, resveratrol exhibits notable cardioprotective potential, particularly through its effects on endothelial function [120]. As the critical interface between circulating blood and vascular tissues, the endothelium plays a pivotal role in maintaining vascular homeostasis [121]. Mechanistic studies suggest resveratrol supports endothelial health via two primary pathways: increasing nitric oxide bioavailability to promote vasodilation and peripheral blood flow, and reducing vascular inflammation, as indicated by lower ICAM-1 levels [120]. These dual mechanisms of enhanced endothelial-dependent vasodilation and anti-inflammatory activity may collectively lower risk of age-related cardiovascular disease. While further work is needed to clarify dosing and long-term efficacy in humans, resveratrol’s multi-target actions support its potential role in healthy aging across multiple systems.

### Magnesium

Magnesium is a naturally occurring mineral found in foods like unrefined whole grains, spinach, nuts, legumes, and potatoes [122]. Epidemiological and clinical evidence demonstrates that higher magnesium intake, whether achieved through dietary sources or supplementation, is associated with a reduced risk of stroke, heart failure, type 2 diabetes, and all-cause mortality [123, 124]. In developed nations, the primary etiology of magnesium deficiency is dietary insufficiency, typically resulting from excessive consumption of processed foods and inadequate intake of magnesium-rich whole foods [123, 125].

Notably, magnesium exhibits a strong positive association with key musculoskeletal metrics, including muscle mass preservation, strength maintenance, and reduced sarcopenia prevalence [126]. This relationship holds clinical relevance given that sarcopenia (age-related muscle loss and function) represents an inevitable physiological process affecting all aging individuals [127]. Wang et al. [128] conducted a systematic review and meta-analysis of 14 randomized controlled trials examining magnesium supplementation’s effects on muscle fitness. Their findings indicate that while magnesium supplementation demonstrates measurable benefits for individuals with magnesium deficiency, particularly older adults, it shows limited efficacy in populations with adequate baseline magnesium status. Magnesium supplementation may serve as a potential preventative strategy against age-related pathologies, supported by its established roles in cardiovascular and metabolic homeostasis, as well as muscle function. However, clinical efficacy appears dependent on baseline nutritional status and may be most beneficial for individuals with documented deficiency. Overall, magnesium plays an important role in cardiovascular, metabolic, and musculoskeletal health, with supplementation appearing most beneficial for individuals with inadequate dietary intake or magnesium deficiency.

### Alpha-Lipoic acid

Alpha-lipoic acid (ALA) is a bioactive molecule that functions as a potent antioxidant [129]. It has garnered substantial research interest due to its dual roles as a mitochondrial antioxidant and metabolic modulator [130]. ALA exerts antioxidant effects within mitochondria, supporting cellular function under conditions of oxidative stress [131].

Emerging evidence suggests ALA supports mitochondrial biogenesis via regulation of PGC-1 signaling pathways [132], Similarly, these processes are implicated in tissues with high energetic demand, including skeletal muscle and neural tissue. In aging and metabolically stressed cells, elevated oxidative stress can impair PGC-1α activity, limiting mitochondrial renewal. ALA helps mitigate this suppression, thereby supporting mitochondrial function and metabolic health [132]. This effect appears particularly relevant in skeletal muscle and neural tissues, where mitochondrial turnover is critical for function. The metabolic benefits of ALA are well-established across multiple clinical populations. A comprehensive 2022 meta-analysis of 16 randomized controlled trials observed significant effects of ALA supplementation on levels of cardiometabolic risk factors [130]. These findings were supported by a separate 12-week randomized controlled trial which reported that daily supplementation with 600 mg of ALA significantly improved lipid profiles in metabolic syndrome patients [21].

The unique dual functionality of ALA positions it as a particularly promising therapeutic intervention for age-related conditions. Specifically, ALA addresses two key hallmarks of aging, mitochondrial dysfunction and insulin resistance [130–132]. This multifaceted mechanism of action suggests ALA may be particularly valuable for managing the complex pathophysiology of age-related metabolic disorders, though optimal dosing strategies remain an active area of investigation. Supplementation with omega-3 fatty acids, resveratrol, magnesium, and ALA interconnected pathways relevant to cardiometabolic health, including endothelial function, mitochondrial bioenergetics, and glucose and lipid metabolism. While optimal dosing and population-specific effects require further research, these compounds represent promising adjunctive strategies for promoting long-term cardiometabolic wellness.

## Joint, Skin, and Connective Tissue Health

### Collagen Peptides (Types I and III)

Collagen, the most abundant protein in the human body, is a key structural component of muscle, tendons, ligaments, and cartilage. Type I collagen provides tensile strength to connective tissues, whereas Type III collagen contributes elasticity and flexibility, supporting tissue resilience under mechanical stress[133–137]. Collagen synthesis declines with age, leading to reduced tendon and ligament integrity, decreased cartilage function, and impaired musculoskeletal performance [132, 133].

### Collagen Supplementation and Joint Health

Oral supplementation with hydrolyzed collagen peptides (5–10 g/day) has been shown to support joint function, reduce age-related pain, and improve mobility, particularly in older adults with osteoarthritis or activity-related joint stress[138]. Clinical studies demonstrate that collagen peptides may stimulate extracellular matrix synthesis in cartilage, downregulate pro-inflammatory cytokines (IL-1β, TNF-α), and suppress matrix metalloproteinases, collectively promoting tissue repair and functional resilience[138, 139].

### Collagen and Tendon/Ligament Adaptation

Collagen supplementation can also enhance tendon and ligament integrity, particularly when combined with mechanical loading such as resistance training. Trials in adults aged 40–70 years show that 5–10 g/day of specific collagen peptides increases tendon cross-sectional area and stiffness, supporting injury prevention and functional performance in aging populations[138, 139]. These interventions target structural hypertrophy and connective tissue resilience, key determinants of healthspan and musculoskeletal independence.

### Absorption and Dosage Considerations

Hydrolyzed collagen peptides are enzymatically broken down into di- and tripeptides (e.g., Pro-Hyp, Gly-Pro-Hyp), which are efficiently absorbed and preferentially accumulate in connective tissues [133, 134]. Timing supplementation around resistance or load-bearing exercise may further enhance tissue-specific benefits [134, 138, 140].

**Table:** 

Recommended Functional-Aging Focused Dosages			
Health Focus	Optimal Dose	Form	Duration
Joint support (OA, aging adults)	5–10 g/day	Hydrolyzed bovine or porcine collagen peptides	12–24 weeks
Tendon and ligament repair	5 g/day	Specific collagen peptides (e.g., TENDOFORTE^®^)	≥ 12 weeks, with exercise

## Other Potential Anti-Aging Supplements

Other “anti-aging” compounds, including curcumin and urolithin A, show promise due to their antioxidant and anti-inflammatory effects [141, 142]. In postmenopausal women, 150 mg/d curcumin for 8 weeks combined with exercise reduced aortic systolic blood pressure [30]. In healthy adults, both 500 mg/d and 6 g/d curcumin for 7 days increased plasma antioxidant capacity (24% and 20%, respectively), with the lower dose also reducing serum cholesterol and triglycerides [31]. Collectively, evidence suggests curcumin may improve cardiometabolic and oxidative stress biomarkers, as well as markers of cellular damage [141, 143, 144].

There has been an emergence of data on the potential application the urolithin A in aging [142]. Urolithin A is a metabolite produced following gut microflora digestion of polyphenol compounds ellagitannin and ellagic acid [142]. Moreover, urolithin A is a known inducer of mitophagy, a process that refers to the body’s removal of dysfunctional mitochondria [145], and, importantly, urolithin A has been shown to play a role in the degradation of defective mitochondria [142]. Given this mechanism of action, nutritional interventions with urolithin A have gained attention with the theory that consumption of this nutrient would trigger autophagy and apoptosis, potentially deleterious effects of aging [146]. To date, most studies have been performed in human cell lines, showing promise in application areas, such as cardiovascular, musculoskeletal-related, and neurodegenerative diseases [142], with limited human clinical trials. For instance, 1,000 mg of urolithin A for 4 months among 66 older adults improved muscle endurance and walking distance covered while having a decrease in acylcarnitine, ceramides, and C-reactive protein levels [22]. These findings demonstrate that urolithin A can improve aspects of one’s physical ability while improving biomarkers of cardiometabolic health/disease risk.

Chlorella-derived supplements may support multiple functional domains relevant to aging populations, including mitochondrial energetics, metabolic regulation, and immune resilience. In a randomized crossover trial in adults aged 40–75 years, Chlorella growth factor (CGF) supplementation over 90 days improved antioxidant status and reduced markers of oxidative aging, alongside hepatoprotective effects, suggesting enhanced systemic resilience to age-related oxidative stress [26]. In a separate crossover study, chlorella intake altered gut-derived metabolites, increasing fecal dicarboxylic acids and restoring propionate levels in older individuals with low baseline concentrations, indicating potential benefits for metabolic regulation and gut–immune signaling [147]. Collectively, these findings support chlorella-based interventions as a promising, though still emerging, nutritional strategy to support healthy aging across metabolic and immune-related functional pathways.

Emerging evidence suggests spirulina may support multiple functional domains relevant to healthy aging, including metabolic regulation, oxidative stress balance, and immune resilience. In clinical and adult populations, spirulina supplementation (500–1000 mg/day) has been associated with improvements in metabolic and redox regulation, including enhanced antioxidant status and lipid profiles alongside reductions in systemic oxidative stress [29, 148]. In broader adult and older adult populations, spirulina has been associated with improved gut microbiota composition, enhanced antioxidant defenses, and reduced inflammatory markers, supporting its potential role in mitigating inflammation processes [149]. More broadly, recent evidence from a 2025 systematic review and meta-analysis of 22 randomized controlled trials (*n* = 5,385) indicates that spirulina significantly reduces inflammatory markers (CRP, IL-6, TNF-α) and lipid peroxidation (MDA), while increasing total antioxidant capacity across diverse adult populations, including older adults [148]. Earlier meta-analytic findings further support modest improvements in antioxidant enzyme activity and reductions in select inflammatory biomarkers, although effects may vary by population and baseline health status. Further, spirulina supplementation has also been shown to enhance immune function, including increased natural killer cell activity, suggesting attenuation of immunosenescence [150]. Collectively, these findings position spirulina as an emerging functional food with multi-system relevance to aging, particularly across metabolic, inflammatory, and immune domains. Curcumin, chlorella, spirulina, and urolithin A are emerging supplements with potential anti-aging effects, primarily through antioxidant, anti-inflammatory, antidiabetic, and lipid-lowering mechanisms. While the scientific rationale behind these nutrients is strong, limited data are present, with the most compelling data supporting *Chlorella*.

## Conclusions

A growing body of evidence supports the use of specific dietary supplements to promote healthy aging by targeting key physiological systems, including muscle mass, mitochondrial function, cognitive health, and cardiometabolic resilience. Among the available interventions, protein and collagen supplementation currently have the strongest and most consistent evidence, particularly when combined with resistance training, to preserve lean mass, improve strength, and support skin and joint integrity in older adults. In contrast, evidence for antioxidant compounds such as carotenoids, vitamin E, selenium, and quercetin remains mixed and context-dependent, with context-dependent effects on functional outcomes across populations and dosing strategies. Safety, dosage, and individual variability, especially in populations with differing nutritional status, remain key considerations. Limitations across the reviewed studies include heterogeneity in study design, variability in supplement formulations and dosing, short intervention durations, and limited inclusion of older adults with comorbidities or frailty, populations that may benefit most. Additionally, mechanistic insights are often extrapolated from animal or cell models rather than confirmed through human trials, underscoring the need for caution when translating these findings into clinical recommendations. Clinicians and older adults should therefore approach supplementation using a personalized and evidence-informed framework that integrates diet quality, exercise, and individual health status. Future research should focus on determining optimal dosing strategies and long-term safety for protein, collagen, and mitochondrial-targeted compounds such as GlyNAC and NMN, as well as defining which aging phenotypes (e.g., sarcopenia, metabolic decline, cognitive impairment) derive the greatest benefit. Additional priorities include investigating synergistic effects among multi-nutrient combinations, clarifying the role of probiotics in immune and inflammatory health, and developing standardized, age-specific antioxidant guidelines.

## Data Availability

No datasets were generated or analysed during the current study.
